# Outcome and Complications of Combined Phacoemulsification and 23-Gauge Pars Plana Vitrectomy

**DOI:** 10.1155/2019/7918237

**Published:** 2019-03-17

**Authors:** Selcuk Sizmaz, Ebru Esen, Puren Isik, Burcu Cam, Nihal Demircan

**Affiliations:** Cukurova University, School of Medicine, Department of Ophthalmology, Adana, Turkey

## Abstract

**Background:**

With the advances in surgical tools, simultaneous removal of cataract associated with vitreoretinal disorders is gaining popularity. This combined surgery offers several advantages besides limitations. The aim of this study is to assess the outcome and complications of phacoemulsification combined with pars plana vitrectomy (PPV).

**Patients and Methods:**

In this retrospective review, medical charts of patients undergoing phacovitrectomy for coexisting cataract and various vitreoretinal disorders were analyzed. Patient demographics, retinal diagnosis, visual acuities (VA) in logMAR, intraocular pressure (IOP), intraoperative and postoperative complications were assessed. Clear corneal phacoemulsification and 23-gauge transconjunctival PPV were administered in all cases.

**Results:**

Eighty-four eyes of 64 (76.2%) males and 20 (23.8%) females were enrolled. The average age of patients was 59.5 ± 13.8 (18–81). The average period of follow-up was 7.2 ± 7.5 months (1–36). The vitreoretinal diagnoses were as follows: 28 (33.3%) rhegmatogenous retinal detachment, 23 (27.4%) vitreous hemorrhage, 12 (14.3%) intraocular foreign body, 12 (14.3%) epiretinal membrane, 4 (4.8%) macular hole, 4 (4.8%) tractional retinal detachment, and 1 (1.2%) vitreomacular traction. The most common intraoperative complications were miosis and rupture of the posterior capsule (92.9% and 8.3%, respectively). In 8 (9.5%) cases, there was fibrin in the anterior chamber. Posterior synechia developed in 7 (8.3%) of cases. No severe increase in intraocular pressure was evident.

**Conclusion:**

Phacoemulsification combined with PPV is a safe and efficient way of management in cases where cataract coexists with vitreoretinal pathologies.

## 1. Introduction

Coexistence of cataract and vitreoretinal disorders is not an uncommon condition [1]. This association—expected not only in the elderly, but also in cases with trauma, intraocular foreign body, intraocular inflammation, and metabolic disorders—is somehow challenging for optimum outcome. First of all, cataract—correlated with its grade—obscures visualization of the fundus, troubling surgery. On the contrary, vitrectomy is known to cause cataract progression. Lens touch is a potential risk in vitreoretinal surgery. Finally, the thickened lens limits thorough vitreous base cleanup, which is of utmost importance particularly in cases with proliferative vitreoretinopathy (PVR).

Allowing quick visual recovery, simultaneous removal of the lens together with vitreoretinal surgery seems to be a proper option in cases with coexisting cataract and vitreoretinopathies so far. Presumably, the procedure is prone to alterations in intraoperative and postoperative complications like increased anterior chamber reaction with fibrin deposition, posterior synechia, and increased intraocular pressure (IOP). Up to date, the results of combined phacoemulsification and pars plana vitrectomy (PPV) have been evaluated by numerous reports [2–10]. The combo procedure allows faster recovery, thus enhancing cost-effectivity [11, 12].

The aim of this study was to investigate the results of combined phacoemulsification and PPV (phacovitrectomy) in our patients with coexisting cataract and miscellaneous vitreoretinal disorders.

## 2. Materials and Methods

### 2.1. Study Design

We retrospectively reviewed the clinical records of patients who underwent phacovitrectomy between November 2014 and March 2018 at Cukurova University, School of Medicine, Department of Ophthalmology. The research adhered to the tenets of Declaration of Helsinki. Patients were operated following brief information on the benefits and risks of the procedure; a signed informed consent was obtained from all patients prior to surgery.

### 2.2. Patients

An anonymized analysis was undertaken. The preoperative diagnoses were rhegmatogenous retinal detachment (RRD), vitreous hemorrhage (VH), intraocular foreign body (IOFB), epiretinal membrane (ERM), macular hole (MH), vitreomacular traction (VMT), and tractional retinal detachment (TRD) associated with cataract. The cataract significance was graded by the surgeon; thus, the decision to do cataract surgery depended on the surgeon's preference. Patient demographics, change in visual acuity (VA) and IOP, preoperative and postoperative complications, and anatomical outcome were assessed. Snellen visual acuity was recorded first and then converted to logarithm of the minimal angle of resolution (log MAR) for statistical analysis. The IOP measurements were made with Goldmann's applanation tonometer.

The intraocular lens power was calculated using the SRK-T formula. In case the vitreoretinal pathology did not allow accurate measurements, the fellow eye was measured.

All patients were evaluated prior to surgery and at the 1^st^ day, 1^st^ week, and 1^st^ month of the operation and additionally when necessary. Patients with a follow-up shorter than 1 month or cases in which intraocular lens (IOL) could not be implanted for any reason were excluded. Cases of combined phacoemulsification and silicone oil removal were not included either.

The outcome measures were anatomical and functional success by means of visual acuity, preoperative and postoperative complications. Anterior chamber flare was evaluated according to the perspectives of the Standardization of the Uveitis Nomenclature Working Group [13].

In terms of IOP, for practical purposes, the measurements were grouped in four as <10 mm·Hg, 10–19 mm·Hg, 20–29 mm·Hg, and >30 mm·Hg. The measurements of the baseline visit, 1^st^ day, 1^st^ week, 1^st^ month, and final follow-up visit were analyzed.

### 2.3. Surgical Procedure

All patients underwent simultaneous 23-gauge 3-port pars plana vitrectomy (PPV) and clear corneal incision phacoemulsification under general anesthesia by two experienced surgeons (SS & EE). The surgeries were performed with the Eva vitrectomy system (DORC Inc, Zuidland, the Netherlands). Appropriate mydriasis was obtained with two drops of tropicamide 1% and two drops of phenylephrine 2.5% applied ten minutes apart in one hour preceding surgery. First, a 23-gauge one-step valved trocar was placed in the temporal or lower temporal quadrant 4 mm behind the limbus. This was followed by a routine phacoemulsification procedure, with two horizontal meridian side-port incisions and a 2.75 mm clear corneal incision at the superior quadrant. The anterior chamber was filled with sodium hyaluronate 1.6%, and a 5.5 mm in diameter continuous curvilinear capsulorhexis was created with Utrata forceps. Following hydrodissection and hydrodelineation, phacoemulsification with nucleus chopping and irrigation/aspiration of the lens cortex was performed. Intraocular lens was implanted depending on the status of the lens capsule; in the bag implantation, a single-piece hydrophobic acrylic IOL (AcrySof SA60AT; Alcon Inc, Fort Worth, TX) was allowed if the capsular bag was intact or a 3-piece hydrophobic acrylic IOL (AcrySof MA60AT; Alcon Inc, Fort Worth, TX) was placed in the ciliary sulcus in case of a ruptured posterior capsule. After the IOL implantation, the anterior chamber was left filled with the ophthalmic viscosurgical device (OVD). The side-port incision was sealed with stromal hydration and—if required—the clear corneal incision was sutured with a 10-0 nylon stitch with buried knots. This was followed by the placement of at least 2 trocars (a 3rd one was placed in case a chandelier light was needed) in the upper nasal and upper temporal quadrants 3.5 mm behind the limbus. The vitreoretinal surgery was performed sequentially. Various vitreoretinal procedures depending on the pathology, including core and peripheral vitrectomy, epiretinal, and/or internal limiting membrane peeling, endodiathermy, photocoagulation, intraocular foreign body (IOFB) removal, fluid/air and air/tamponade (silicone oil, gas) exchange, and perfluorocarbon liquids, were used on demand. In case of iatrogenic retinal breaks, endotamponade was particularly used. Then, the OVD in the anterior chamber was washed out. By the end of the procedure, the trocars were removed and the sclerotomy incisions were sutured transconjunctivally with 7-0 polyglactin if necessary. The watertightness of all incisions was checked. A subconjunctival injection of dexamethasone and cefuroxime was administered.

In case of intraoperative miosis, epinephrine (0.001%) was injected into the anterior chamber. If this maneuver failed, iris hooks were placed in four quadrants.

In the postoperative period, the patients were prescribed moxifloxacin drops 5 times daily for 10 days, prednisolone acetate 1% drops hourly, which was tapered after 1 week till the 1^st^ month of the surgery, and topical mydriatics (tropicamide 1%, BID) for a week. In cases with severe anterior segment inflammation, subconjunctival dexamethasone was injected daily until the findings subsided.

A posterior synechia was defined if it was detected at two or more postoperative visits; thus, transient synechia or the ones that resolved following pupillary dilation were not taken into account [4].

### 2.4. Statistical Analysis

Statistical analysis was performed using SPSS software (Version 16.0, SPSS Inc., Chicago, IL, USA). All numerical data were expressed as median values (Minimum-Maximum). For each continuous variable, normality was checked by Kolmogorov–Smirnov and Shapiro–Wilk tests and by histograms. Comparisons between groups were done using Kruskal–Wallis test for the data that was not normally distributed. Pre-post measures data were analyzed using Friedman test and Wilcoxon test. The categorical variables between the groups were analyzed by using the chi-square test or Fisher exact values of *p* < 0.05 were considered significant.

## 3. Results

Eighty-four eyes of 64 (76.2%) males and 20 (23.8%) females were enrolled. The average age of patients was 59.5 ± 13.8 (18–81). The average period of follow-up was 7.2 ± 7.5 months (1–36). There were 47 (56.0%) left and 37 (44.0%) right eyes involved. The vitreoretinal diagnoses were as follows: 28 (33.3%) RRD, 23 (27.4%) VH, 12 (14.3%) IOFB, 12 (14.3%) ERM, 4 (4.8%) MH, 4 (4.8%) TRD, and 1 (1.2%) VMT.

The most common intraoperative phacoemulsification-related complication was miosis which was encountered in 78 (92.9%) of cases. In 10 (11.9%), there was intraoperative need for iris hooks for proper pupil dilation. In the rest, the pupil responded well to intracameral epinephrine (0.001%). The second most common complication was rupture of the posterior lens capsule which was evident in 7 (8.3%) of the cases. Three (3.6%) cases, all of which were trauma cases with IOFB, had preoperative ruptured lens capsule.

In 30 (35.7%) cases, 1000-centistoke silicone oil was used, in 17 (20.2%) cases 5000-centistoke silicone oil, and in 22 (26.2%) cases C_3_F_8_ (13%). No endotamponade was used in 15 (17.9%) cases. The clear corneal incision was sutured in 29 (34.5%) cases, and the sclerotomy incisions were sutured in 67 (79.8%) cases; the sclerotomy incisions were particularly sutured in case of silicone oil tamponade.

No cases of corneal edema obscuring fundus visualization were encountered.

In the early postoperative period, the anterior segment reaction was mild in 72 (85.7%) cases and severe in 12 (14.3%) cases. In 8 (9.5%) cases, there was fibrin in the anterior chamber which resolved with subconjunctival injections of dexamethasone in all cases.

The course of IOP did not significantly differ between the diagnoses (Table 1).

The diagnoses were compared by means of gender and associated intraocular complications in Table 2. There was a significant male preponderance in all diagnostic groups. Capsule-related complications (preoperative and intraoperative tear in the posterior lens capsule) were significantly higher in IOFB cases. The use of intraocular tamponade was significantly higher in RD cases, as expected. The diagnostic groups were not significantly different by means of postoperative reaction or fibrin in the anterior chamber. Suturing the incisions did not significantly differ according to diagnosis either. The endotamponade used did not significantly interfere with the development of anterior chamber cells or fibrin or posterior synechia formation (*p*=0.813, 0.664, 0.333, respectively). Of the 10 cases in which iris hooks were used for pupil dilation, only one ended up with posterior synechia (*p*=0.652).

In 4 (33.3%) ERM cases, silicone oil (1000 cst) endotamponade had to be used due to iatrogenic breaks.

No cases of postoperative hypotony, hyphema, IOL dislocation, toxic anterior segment syndrome (TASS), or endophthalmitis occurred. None of the cases that the clear corneal incision was not sutured revealed a positive Seidel's test on the first postoperative day. No patient developed posterior capsular opacification (PCO) or neovascular glaucoma (NVG).

## 4. Discussion

Cataract accompanying vitreoretinal disorders could result in a surgical challenge. A number of advantages were reported regarding phacovitrectomy. Providing a clear media, it allows better visualization of the fundus. Vitreous base removal has utmost importance particularly in RRD cases and in cases with PVR; thus, the removal of the crystalline lens helps improvement in visualization and shaving of the vitreous base [14]. Moreover, a thickened crystalline lens could easily end up with intraoperative lens touch by vitreoretinal instruments; this condition is avoided by combo surgery. Finally, cataract progression is a well-known complication of PPV. Cataract progression rate was reported to be 80% in the 2-year period after the PPV [15, 16]. Thus, we could easily argue that cataract surgery is almost inevitable following PPV. On the contrary, cataract surgery could be challenging in a previously vitrectomized eye; numerous conditions, like capsular and zonular problems, iris-lens extrapulsion syndrome, were reported; hence, previous studies advocated pars plana vitrectomy with combined phacoemulsification [17, 18]. Combined surgery helps minimizing the economic cost [11]. As a last, but not least advantage, combined surgery presumably allows quicker visual rehabilitation.

On the contrary, combo surgery has several shortcomings. One of the main disadvantages is prolonged surgical duration [7]. The procedure is expected to cause more severe anterior segment inflammation, compared to sequential surgery. Continuous curvilinear capsulorhexis might be challenging when the red fundus reflex is absent due to the retinal pathology, e.g., dense vitreous hemorrhage [14]. The risk of posterior capsule rupture is expected to be increased; hence, combo surgery requires enhanced surgical experience. Moreover, risk of posterior synechia formation is increased with phacovitrectomy [2–4, 14]. One final limitation of the procedure is the vanishing of the accommodative status of the eye. Due to the aforementioned advantages, it would not be a demanding decision to remove the crystalline lens in a presbyopic patient; however, in young patients with accommodative capability, phacoemulsification accompanying PPV would be challenging. The mean age of our patients was 59.5, which was very similar with the finding of another study [9].

We encountered a high rate of intraoperative miosis (92.9%) during phacoemulsification which mostly responded to intracameral epinephrine 0.001%. To the best of our knowledge, no study on this topic reported a particular rate of intraoperative miosis.

Another issue regarding combined surgery is the timing of IOL implantation. Some studies advocated the implantation of the IOL should be performed at the very end of the procedure due to a better visualization of the far periphery of the retina as the edge of the IOL could cause some prismatic effects deteriorating visibility [19, 20]. On the contrary, we—like Jain and co-workers [2] suggested—placed the IOL at the end of phacoemulsification, before PPV. We believe this helps avoiding unintentional capsular damage with the vitrector and preventing a capsular bulge when endotamponades are used.

Leiderman et al. and co-workers [9] reported a 3.3% rate of synechia. In a controlled study, Park et al. and co-workers [21] reported that phacovitrectomy cases revealed significantly higher rates of abnormal IOP (60%), intraocular fibrin (30%), and synechia (30%) compared to PPV-alone cases. The rate of posterior synechia and fibrin was 8.3% and 9.5%, respectively, in total in our group. This difference might be due to the variety in the diagnosis. Moreover, we believe the relatively high rate of (14.3%) IOFB cases in our group might have caused an increase in fibrin and synechia formation. On the contrary, there was no significant difference in terms of anterior chamber inflammation in different diagnostic groups. Perhaps this could be attributed to the relatively aggressive topical steroid therapy, particularly in the very first postoperative week. In a study consisting of various vitreoretinal disorders, IOP rise, fibrin, and synechia rates were reported to be 3.8%, 7.7%, and 5.8%, respectively, which were similar to our rates [2]. Zheng and co-workers [3] reported the aforementioned rates to be 10.8%, 6.3%, and 4.9%, respectively. In Oh and co-workers' [4] study, the posterior synechia rate was 6.1%, and the authors concluded that the use of endotamponade and prolonged operation could be the risk factors. Use of iris hooks could potentially be associated with increased inflammation and posterior synechia due to a broken blood-aqueous barrier. However, in this series, we did not find a significant relationship; perhaps, this could be due to relatively small rate of iris hooks use.

Karaca and co-workers [11] pointed some concerns in phacovitrectomy. These are difficulty in maintaining the anterior chamber depth particularly during scleral indentation, diminished posterior view due to endothelial stria, IOL decentration, and passing of the tamponade into the anterior chamber due to zonular problems. Thus, the authors were in favor of leaving the OVD in the anterior chamber intraoperatively and postoperatively. Of sure, as the authors stated, this could be associated with postoperative complications like rise in IOP, capsular block, and toxic anterior segment syndrome (TASS). Depending on similar concerns, we washed the OVD out of the anterior chamber at the conclusion of the surgery. We did not aim a thorough removal; on the contarry, we did not encounter a serious rise in IOP. At the first day following surgery, 3 (3.6%) patients had IOP values of 21–29 and only 1 (1.2%) patient had an IOP value of over 30 mm·Hg. These were controlled with topical medication. No cases of capsular block or TASS were evident.

Intraoperative corneal edema is not an uncommon complication of combined procedures [14]. Significant corneal edema which would require corneal scraping was not evident in any patient. Perhaps, this is due to the irrigation solution used and the enhanced fluidics of the vitreoretinal surgery machines.

Perhaps, this study has several limitations, its retrospective and uncontrolled nature being the top-ranked. Moreover, we did not enroll the surgical parameters belonging to PPV. However, depending on the increase in visual acuity in each diagnostic group, we could consider that vitreoretinal surgery achieved success. Our group was relatively small, comprising various vitreoretinal disorders. Hence, it would end up with conflicting data if we did involve the vitreoretinal outcome. In this manuscript, we sought to analyze particularly the intraoperative and postoperative anterior segment findings, complications. Nevertheless, a study comparing the outcomes of combo surgery and solely PPV in patients with cataract and associated vitreoretinal disorders would have provided more precise findings. The retrospective design might have also caused some intraoperative findings like iris prolapse, iris damage, or hemorrhage in the anterior chamber to be overlooked.

In conclusion, combined phacoemulsification and PPV for coexisting cataract and vitreoretinal pathologies yield favorable outcome. We believe the risks are opposed by benefits.

## Figures and Tables

**Table 1:** The change in intraocular pressure according to diagnosis.

	Diagnosis					
	RD	VH	IOFB	ERM	Others	*p*
	*n* (%)	*n* (%)	*n* (%)	*n* (%)	*n* (%)	
*Baseline*						
<10	2 (7.1)	0	1 (8.3)	1 (8.3)	0	0.763
10–19	26 (92.9)	21 (91.3)	10 (83.3)	10 (83.3)	9 (100)	
20–29	0	1 (4.3)	1 (8.3)	1 (8.3)	0	
>30	0	1 (4.3)	0	0	0	

*1* ^*st*^ *day*						
<10	2 (7.1)	0	3 (25.0)	5 (41.7)	0	0.070
10–19	11 (39.3)	12 (52.2)	6 (50.0)	3 (25.0)	4 (44.4)	
20–29	6 (21.4)	3 (13.0)	1 (8.3)	1 (8.3)	1 (11.1)	
>30	9 (32.1)	8 (34.8)	2 (16.7)	3 (25.0)	4 (44.4)	

*1* ^*st*^ *week*						
<10	2 (7.1)	0	0	0	1 (11.1)	0.510
10–19	19 (67.9)	19 (82.6)	12 (100)	11 (91.7)	5 (55.6)	
20–29	5 (17.9)	3 (13.0)	0	1 (8.3)	2 (22.2)	
>30	2 (7.1)	1 (4.3)	0	0	1 (11.1)	

*1* ^*st*^ *month*						
<10	1 (3.6)	1 (4.3)	0	0	0	0.594
10–19	24 (85.7)	21 (91.3)	11 (91.7)	10 (83.3)	8 (88.9)	
20–29	3 (10.7)	0	1 (8.3)	2 (16.7)	0	
>30	0	1 (4.3)	0	0	1 (11.1)	

*Final visit*						
<10	0	0	0	1 (8.3)	0	0.182
10–19	26 (92.9)	22 (95.7)	12 (100)	10 (83.3)	8 (88.9)	
20–29	2 (7.1)	1 (4.3)	0	1 (8.3)	0	
>30	0	0	0	0	1 (11.1)	

RD: retinal detachment; VH: vitreous hemorrhage; IOFB: intraocular foreign body; ERM: epiretinal membrane; others: macular hole, tractional retinal detachment, and vitreomacular traction. Chi-square test.

**Table 2:** Gender, intraoperative, and postoperative parameters according to the diagnosis.

	Diagnosis					
	RD	VH	IOFB	ERM	Others	*p*
	*n* (%)	*n* (%)	*n* (%)	*n* (%)	*n* (%)	
*Gender*						
Male	24 (85.7)	16 (69.6)	12 (100)	8 (66.7)	4 (44.4)	**0.023**
Female	4 (14.3)	7 (30.4)	0	4 (33.3)	5 (55.6)	

*Lens capsule*						
Intact	28 (100)	21 (91.3)	6 (50.0)	10 (83.3)	9 (100)	**0.0001**
Intraoperative rupture	0	2 (8.7)	3 (25.0)	2 (16.7)	0	
Preoperative rupture	0	0	3 (25.0)	0	0	

*Iris hooks*						
None	23 (82.1)	21 (91.3)	9 (75.0)	12 (100)	9 (100)	0.201
Present	5 (17.9)	2 (8.7)	3 (25.0)	0	0	

*Corneal suture*						
None	18 (64.3)	17 (73.9)	4 (33.3)	10 (83.3)	6 (66.7)	0.094
Present	10 (35.7)	6 (26.1)	8 (66.7)	2 (16.7)	3 (33.3)	

*Scleral suture*						
None	7 (25.0)	3 (13.0)	2 (16.7)	4 (33.3)	1 (11.1)	0.564
Present	21 (75.0)	20 (87.0)	10 (83.3)	8 (66.7)	8 (88.9)	

*Endotamponade*						
None	0	7 (30.4)	3 (25.0)	5 (41.7)	0	**0.011**
S. O. 1000	13 (46.4)	6 (26.1)	6 (50.0)	4 (33.3)	1 (11.1)	
S. O. 5000	9 (32.1)	5 (21.7)	1 (8.3)	0	2 (22.2)	
Gas	6 (21.4)	5 (21.7)	2 (16.7)	3 (25.0)	6 (66.7)	

*Posterior synechia*						
Absent	27 (96.4)	21 (91.3)	12 (100)	11 (91.7)	6 (66.7)	0.054
Present	1 (3.6)	2 (8.7)	0	1 (8.3)	3 (33.3)	

*Fibrin*						
None	25 (89.3)	21 (91.3)	11 (91.7)	11 (91.7)	8 (88.9)	0.998
Present	3 (10.7)	2 (8.7)	1 (8.3)	1 (8.3)	1 (11.1)	

*Anterior chamber cells*						
Mild	14 (50.0)	11 (47.8)	4 (33.3)	6 (50.0)	5 (55.6)	0.948
Moderate	10 (35.7)	8 (34.8)	7 (58.3)	4 (33.3)	3 (33.3)	
Severe	4 (14.3)	4 (17.4)	1 (8.3)	2 (16.7)	1 (11.1)	

RD: retinal detachment; VH: vitreous hemorrhage; IOFB: intraocular foreign body; ERM: epiretinal membrane; others: macular hole, tractional retinal detachment, and vitreomacular traction. Chi-square test.

## Data Availability

The Excel data used to support the findings of this study are available from the corresponding author upon request.
